# Comparative Analysis of Methods to Induce Myocardial Infarction in a Closed-Chest Rabbit Model

**DOI:** 10.1155/2015/893051

**Published:** 2015-10-04

**Authors:** Marc-Antoine Isorni, Amaury Casanova, Julie Piquet, Valérie Bellamy, Charly Pignon, Etienne Puymirat, Philippe Menasche

**Affiliations:** ^1^Department of Cardiology, Hôpital Européen Georges Pompidou, Assistance Publique des Hôpitaux de Paris, France; ^2^Université Paris Descartes, Paris, France; ^3^INSERM U970, Paris Cardiovascular Research Center PARCC, Paris, France; ^4^National Veterinary School of Alfort, Maisons-Alfort, France; ^5^Department of Cardiovascular Surgery, Hôpital Européen Georges Pompidou, Assistance Publique des Hôpitaux de Paris, France

## Abstract

*Objective.* To develop a rabbit model of closed-chest catheter-induced myocardial infarction.* Background.* Limitations of rodent and large animal models justify the search for clinically relevant alternatives.* Methods.* Microcatheterization of the heart was performed in 47 anesthetized 3-4 kg New Zealand rabbits to test five techniques of myocardial ischemia: free coils (*n* = 4), interlocking coils (*n* = 4), thrombogenic gelatin sponge (*n* = 4), balloon occlusion (*n* = 4), and alcohol injection (*n* = 8). In order to limit ventricular fibrillation, an antiarrhythmic protocol was implemented, with beta-blockers/amiodarone before and xylocaine infusion during the procedure. Clinical, angiographic, and echographic data were gathered. End points included demonstration of vessel occlusion (TIMI flow grades 0 and 1 on the angiogram), impairment of left ventricular function at 2 weeks after procedure (by echocardiography), and pathologically confirmed myocardial infarction.* Results.* The best arterial access was determined to be through the right carotid artery. The internal mammary guiding catheter 4-Fr was selected as the optimal device for selective intracoronary injection. Free coils deployed prematurely and tended to prolapse into the aorta. Interlocking coils did not deploy completely and failed to provide reliable results. Gelatin sponge was difficult to handle, adhered to the catheter, and could not be clearly visualized by fluoroscopy. Balloon occlusion yielded inconsistent results. Alcohol injection was the most efficient and reproducible method for inducing myocardial infarction (4 out of 6 animals), the extent of which could be fine-tuned by using a coaxial balloon catheter as a microcatheter (0.52 mm) to achieve a superselective injection of 0.2 mL of alcohol. This approach resulted in a 20% decrease in LVEF and infarcted myocardium was confirmed histologically.* Conclusions.* By following a stepwise approach, a minimally invasive, effective, and reproducible rabbit model of catheter-induced myocardial infarction has been developed which addresses the limitations of rodent experiments while avoiding the logistical and cost issues associated with large animal models.

## 1. Introduction

Heart failure (HF) is a major health and economic burden in developing countries and its prevalence is increasing [1, 2]. It is a complex, heterogeneous disorder that can result from primary cardiomyopathy or, more commonly, from myocardial infarction (MI), hypertension, or disorders of the valve [3, 4]. Because many HF cases are secondary to ischemic heart disease, animal models that closely mimic the characteristics and development of human MI are essential for better understanding the pathophysiology of heart failure and for developing medical or surgical therapies. Many of these models entail a surgical ligation of the coronary artery (with or without reperfusion), which is a reasonably reproducible technique, with the caveat that it results in a HF pattern different from what is seen in humans [5–7]. Percutaneous intracoronary embolization of various materials more closely duplicates acute coronary syndromes caused by embolization of atherosclerosis debris or thrombosis in the coronary microcirculation. Furthermore, the minimally invasive nature of the procedure reduces surgical complications and maintains the integrity of anatomical structures, particularly the pericardium which is involved in the regulation of myocardial inflammation and remodeling [6, 8–13]. However, the embolization technique also has limitations. First, it may be challenging to precisely control the location and duration of the coronary artery occlusion. Second, so far, it has been primarily applied to large animals (specifically pig and sheep). In an attempt to find an acceptable trade-off between rodent models, whose cardiac physiology is far from that of humans [14], and large animal models, which raise logistical and economic issues, the present study was designed to develop a straightforward and reproducible model of percutaneously induced MI in rabbits.

## 2. Materials and Methods

### 2.1. Animals

This study was approved by the University of Paris René Descartes Ethics Committee and by the* ad hoc* board of the French Ministry of Research and Education. Animals were cared for in accordance with the Guide for the Care and Use of Laboratory Animals [15].

### 2.2. Experimental Design

Adult male White New Zealand rabbits weighing 3.8–4.4 kg were used in this study. During the procedure, animals were anaesthetised using 5 mg/kg ketamine-HCl and 20 mg/kg propofol via the ear vein and anticoagulated with heparin (300 IU/kg). An antiarrhythmic protocol was implemented, which included beta-blockers/amiodarone and lidocaine (1 mg/kg load followed by a 20 *μ*g/kg/min infusion) to reduce ischemic ventricular arrhythmias. Electrocardiogram and peripheral oxygen saturation were continuously recorded. Procedure success was assessed by the demonstration of a widening of the QRS complex or of ST segment elevation in the electrocardiogram and by TIMI 0 or 1 angiographic blood flow grades. After induction of MI, animals were kept under clinical observation. Animals with postprocedural heart failure received furosemide at 0.3 mg/kg/d for 3 days. After 3 weeks, animals were sedated as described above, examined clinically and echographically, and sacrificed. End points included impairment of left ventricular (LV) function at 3 weeks after procedure (by echocardiography using the Simpson method) and histologically confirmed myocardial infarction.

### 2.3. Experimental Procedures

Three series of experiments were sequentially performed.

(1) Assessment of vascular access and coronary catheterization procedures: after having selected the right carotid artery as the vascular access entry site, 3 rabbits were used to test two devices (cathlons and fine needles); 2 animals were used for vessel catheterization and to select the optimal introducer sheath size. Four additional rabbits were then used for selecting the type and size of the guiding catheter for coronary catheterization and to determine the dilution of the contrast medium.
(2) Assessment of the coronary occlusion site: to determine the coronary artery branch most suitable for occlusion to induce a functionally relevant MI, 14 rabbits underwent release of free and droppable coils. They were divided into three groups according to the target territory: the left descending artery area (*n* = 6), the circumflex artery territory (*n* = 5), or the right coronary artery territory (*n* = 3). After three weeks, sedated animals were evaluated clinically and echographically, according to the protocol described above, before being sacrificed and studied histologically.
(3) Comparison of MI-inducing techniques: five different techniques of coronary occlusion were finally investigated: free coils (*n* = 4), interlocking coils (*n* = 4), balloon occlusion (*n* = 4), release of a thrombogenic sponge (*n* = 4), and injection of alcohol (*n* = 8).

For coils, a guiding catheter was positioned under fluoroscopic guidance at the coronary ostium and a 0.014-in. floppy wire was advanced into the coronary vessel. The wire was advanced into the dominant coronary artery branch, as determined by angiography (Figure 3). Over the wire, a microcatheter (FineCross MG, Terumo Corporation, Tokyo, Japan) was tracked into the coronary artery. Free coils were loaded into the microcatheter and dropped to the desired site. For interlocking coils, the procedure was modified in that the microcatheter was tracked, the floppy wire was removed, and the interlocking coil was loaded into the inner lumen of the microcatheter. It was then advanced to the optimal site and deployed. If interlocking coils failed to deploy, they could be removed. For balloon occlusion, an angioplasty balloon was tracked over the wire, placed into the target vessel, and inflated to occlude it. The desired period of occlusion was 300 seconds. In the thrombogenic sponge-injected group, a collagen sponge (Gelfoam, Pfizer, New York, NY, USA) was cut according to the size of the target vessel, impregnated with a contrast solution and dropped through the microcatheter. For alcohol injection, the microcatheter had to be positioned so as to occlude the coronary artery. When a lack of retrograde flow was confirmed by angiography, the alcohol was injected into the microcatheter. Because of insufficient selectivity, we used a coaxial total chronic coronary occlusion balloon catheter as a microcatheter to achieve a superselective injection of 0.2 mL of alcohol (Mini-Trek, Abbott, Chicago, IL, USA). This device offers a better crossing profile and a better push. After removal of the 0.014-in. floppy wire, contrast medium was injected into the inner lumen of the balloon to verify vascular occlusion. Alcohol was then injected into the target artery to induce necrosis of the adjacent tissue and hence induce an infarction.

### 2.4. Histological Examination

**Figure 1:**
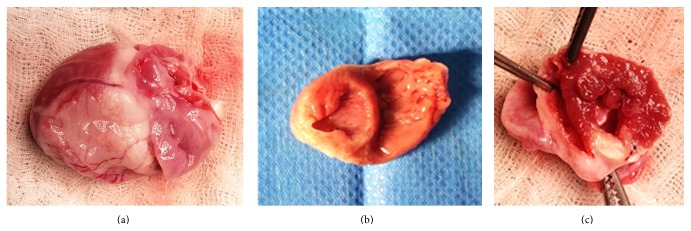
Macroscopic views. Macroscopic views of a heart that underwent embolization of the middle segment of the right coronary artery; rear side view shows myocardial scarring of the right ventricle and the inferior wall of the left ventricle (a). The area of fibrosis is the inferior wall of the left ventricle and right ventricle (b). Heart that underwent embolization of the middle segment of the circumflex artery; apical section shows lateral wall transmural myocardial infarction (c).

**Figure 2:**
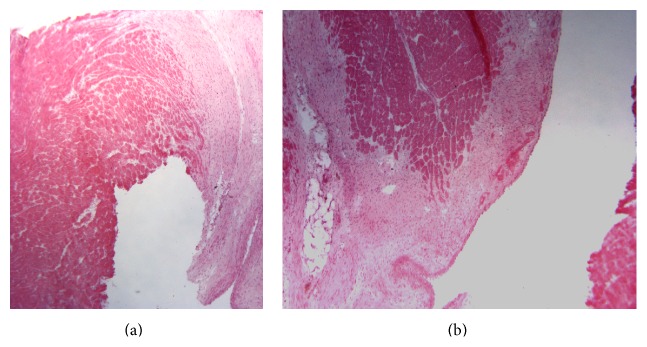
Histological analysis of left ventricle lateral wall transmural infarction. Histological views of left ventricle lateral wall infarction focused on left ventricle anterior wall after mid segment circumflex artery alcohol injection (a) and of the interventricular septum focused on the basal segment after proximal segment right coronary artery alcohol injection (b) showing muscle and fibrosis. Heart was excised, cut, then embedded in OCT, flash frozen in liquid nitrogen, and stored at −80°C. Ten-micrometer cryosections were stained to confirm the presence of ischemic tissue.

**Figure 3:**
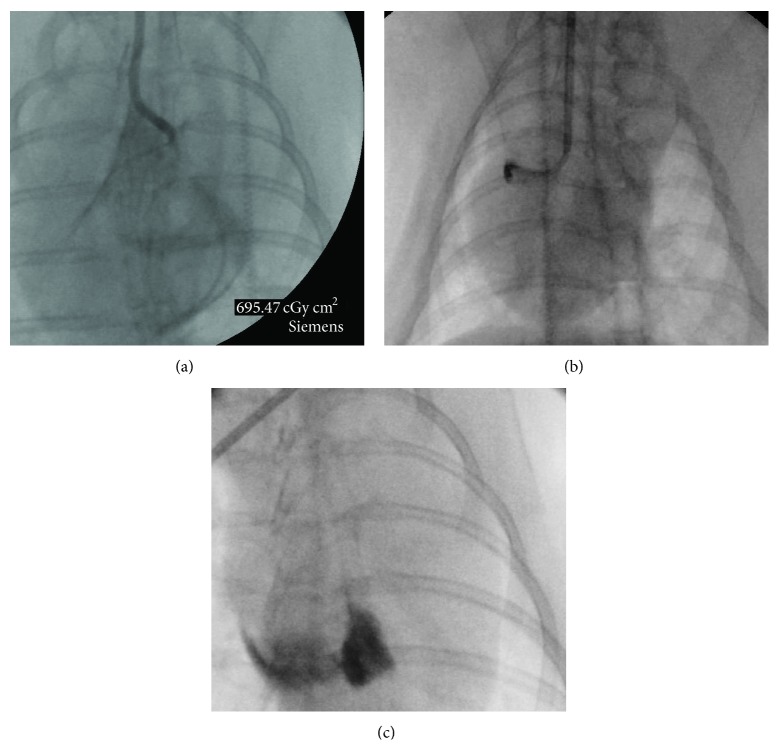
Angiography of the coronary artery after mid segment alcohol injection. Selective angiography of the left main coronary artery, of the left anterior descending along the anterior interventricular sulcus reaching the apex of the heart, of a diagonal coronary artery, and of the circumflex coronary artery along the atrioventricular groove (a). Embolization of the right coronary artery ostium with free coil (b). Angiography of the right coronary artery after mid segment alcohol injection (c). The contrast product injected after alcohol injection stagnates in the necrotic area thus achieving an aspect of myography.

Hearts in which a myocardial infarct was macroscopically observed at autopsy underwent a histological analysis (Figures 1 and 2). Hearts were excised, cut in the coronal plane into three sections from apex to base, embedded in OCT, flash-frozen in liquid nitrogen, and stored at −80°C. Ten-micrometer cryosections were stained with hematoxylin-eosin to confirm the presence of ischemic tissue.

### 2.5. Echocardiography

*In vivo* heart function was evaluated by echocardiography three weeks after ischemic injury. Transthoracic echocardiography was performed on sedated animals by an experienced cardiologist using an echocardiography machine equipped with an appropriate probe for imaging rabbit hearts (Vivid E9 ultrasound platform, GE Health Care, Pittsburgh, PA, USA). Two-dimensional mages were taken using both long axis and short axis views. Measurements were taken to evaluate the following parameters: dimensions of the LV, left atrium, wall thickness, valve function, and LV ejection fraction (calculated using Simpson's method).

### 2.6. Statistical Analysis

Data are given in percentages and means ± standard deviations. Comparisons between groups were made using the nonparametric ANOVA test for continuous variables. A *p* < 0.05 value was considered statistically significant. Statistical analysis was performed with JMP 9.1 software (SAS, Cary, NC, USA).

## 3. Results

### 3.1. Assessment of Vascular Access and Coronary Catheterization Procedures

The safest micropuncture vascular access was achieved using needles and 0.018-in. wires. A 4-Fr micropuncture sheath was effective. It was advanced over the wire and exchanged for a 0.035-in. guidewire to support the placement of a 4-Fr internal mammary guide catheter (Cordis Corporation, Miami, FL, USA), with custom alteration for use in a rabbit. The guiding catheter was advanced retrogradely to the ascending aorta using fluoroscopy, and an angiography of the coronary artery was then performed to check for its adequate location. Dilution of the contrast medium was kept at 50% of the total volume to reduce the risk of ventricular arrhythmias.

### 3.2. Assessment of the Coronary Occlusion Site

Middle or proximal segment occlusion of the left anterior descending coronary artery led to severe heart failure with massive hemorrhagic infarction of the LV, while distal segment occlusion caused severe conduction disturbances requiring isoprenaline infusion. The occlusion of the branches (diagonal and septal branches) needed a custom device and led to only small nontransmural infarcted areas, without a relevant decrease in LVEF. Occlusion of obtuse marginal branches yielded a very limited, nontransmural LV infarction without functional consequences, as assessed by echocardiography. Only occlusion either of the mid segment of the right coronary artery or of the mid segment of the circumflex coronary artery was finally found to be the most reliable procedure for inducing an extensive infarction of the supplied LV area. Such infarcts led to an akinesia/hypokinesia of the right ventricle and of the inferior wall of the left ventricle (right coronary occlusion or circumflex coronary occlusion, resp.) with, in both cases, a 25% decrease in LVEF and only nonsustained ventricular arrhythmias. These data (summarized in Table 1) led to the conclusion that, according to the coronary artery distribution, the dominant vessel would be the best target for embolization.

### 3.3. Comparison of MI-Inducing Techniques

#### 3.3.1. Free Coil Embolization

Four rabbits underwent free coil embolization. Two coils deployed at the left main artery ostium and protruded into the aorta. One coil deployed at the circumflex artery proximal segment and one deployed at the right coronary artery ostium. All of these rabbits died from intraoperative refractory ventricular fibrillation. Histological examination revealed large left and right ventricle hemorrhagic necrosis, depending on the occlusion site (Tables 2 and 3).

#### 3.3.2. Interlocking Coil Embolization

Four rabbits underwent interlocking coil embolization in the mid segment of the target coronary artery. Two animals died from intraoperative refractory ventricular fibrillation during coil deployment in the circumflex and right coronary arteries, possibly due to the unwanted occlusion of the more proximal arterial segment due to excessively long coils. Two animals survived the procedure and until the end of the study. In both of these cases, the coils were too short, causing their migration towards the distality of the target vessel (the circumflex artery in one case and the right coronary artery in the other). Expectedly, this resulted in limited nontransmural or even undetectable infarct areas without changes in LVEF (Tables 2 and 3).

#### 3.3.3. Gelatin Sponge Embolization

**Figure 4:**
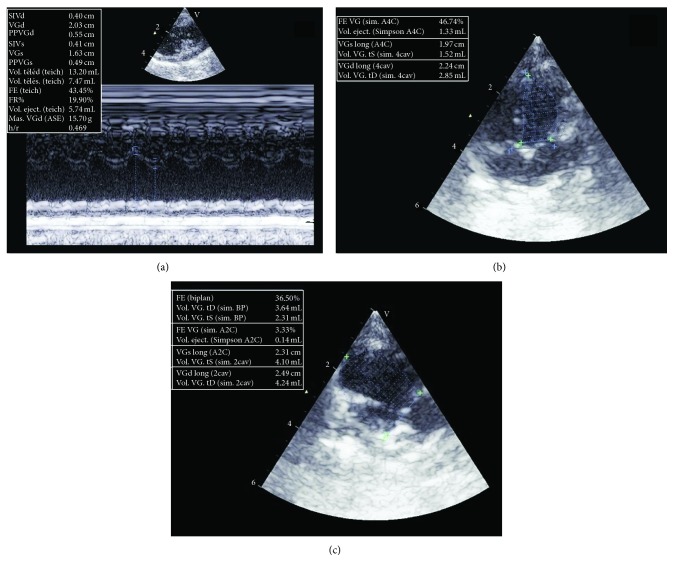
Echographic assessment of left ventricular ejection fraction. Left ventricular measurements are made with the M-mode. Both ventricular diameters (systolic and diastolic) are measured from the leading edge to leading edge of each interface, and LVEF by Teichholz is then calculated (a). Ventricular volumes are calculated in the two orthogonal apical views: four-chamber (b) and (c)* biplane Simpson*. Left ventricular endocardium is traced in end-diastole and end-systole in both views.

Gelatin sponges were used in four procedures (three right coronary artery mid segment embolization and one circumflex mid segment embolization). No intraoperative or postoperative arrhythmias occurred and all animals survived until the end of the study. The likely reason was that only histologically limited nontransmural LV lateral wall infarcts were detectable after circumflex embolization while no myocardial damage could be identified in the case of right coronary artery embolization. No LVEF decrease could be echographically detected in any of these cases (Figure 4; Tables 2 and 3).

#### 3.3.4. Balloon Occlusion

Four animals underwent balloon occlusion, of the right coronary mid segment (two cases) and of the circumflex mid segment (two remaining cases). All animals survived despite the occurrence of nonsustained ventricular tachycardia which resolved after balloon deflation. Both echocardiographic and histological examinations failed to reveal any cardiac abnormality (Tables 2 and 3).

#### 3.3.5. Alcohol Injection

Eight animals underwent alcohol injection in the mid segment of the right coronary artery and of the circumflex artery in five and three cases, respectively. All angiograms performed after alcohol injection showed coronary artery TIMI flow 0 or I and a pattern of myography in the infarcted area. One animal with circumflex artery alcoholization died from refractory ventricular fibrillation. Another rabbit which had undergone alcohol injection in the right coronary artery died two days after surgery from drug-refractory heart failure. However, the 6 remaining rabbits survived until the end of the study. Four of them were then found to have a macroscopically massive infarction of the right ventricle and of the LV inferior wall, which was confirmed by histological analysis. Two other rabbits had massive infarction of the left ventricle lateral wall. In all of these survivors, LVEF was significantly reduced from its baseline value (19,4 ± 2,4%; Tables 2 and 3).

## 4. Discussion

It is generally accepted that proof-of-concept studies designed to test the efficacy of new drug-, cell-, or other biologics-based therapies for HF can be reliably performed in rat and mouse [16, 17]. However, the drawbacks of these rodent models are also well recognized. They primarily include major differences in heart physiology compared with humans and a limited lifespan which precludes long-term follow-up assessments. These hurdles can be overcome by the use of large animal models, primarily pig and sheep, which offer distinct advantages: a more clinically relevant cardiac physiology, a size allowing testing catheter-based delivery devices, and the possibility of realistically studying dose-effect relationships [14, 18, 19]. However, these models are more logistically complex and costly as they require dedicated facilities and highly trained personnel. In this setting, the rabbit appears as an attractive trade-off. The physiology of this species is not so far from that of humans with regard to heart rate and patterns of contractility-related calcium handling and myosin heavy chain isoforms, while vessels size is compatible with the testing of catheter-based interventions [20, 21]. Indeed, several studies have used a rabbit model of MI induced by permanent or transient coronary artery ligation, but, in most cases the procedure has been performed through a direct surgical approach of the target artery [22]. Closed-chest procedures have been less commonly reported despite distinct advantages such as a limited invasiveness and the possibility of keeping the pericardium untouched and free from adhesions. The latter avoids the confounding effect of pericardial incision on postinfarction remodeling. This model also allows, when indicated, a subsequent direct intramyocardial delivery of the product under investigation [5, 23]. The recognition of these advantages prompted the present study which entailed the first angiography-based characterization of the coronary artery anatomical patterns in rabbits and the development of a reproducible percutaneous catheter-based technique of myocardial necrosis induction.

Our review of the angiograms that we performed in our initial series of 14 rabbits did not yield results which matched those previously published [24, 25]. Basically, the coronary artery anatomy of the rabbit seems to be organized as in humans with a constant left main coronary artery and left or right coronary artery dominance depending on whether the posterior descending artery originates from the circumflex coronary artery or the right coronary artery, respectively. The left main artery divides into the left anterior descending (LAD) and the circumflex arteries. The LAD travels in the anterior interventricular groove and continues up to the apex of the heart. It supplies the anterior part of the septum with septal branches and the anterior wall of the left ventricle with diagonal branches. The circumflex and right coronary arteries supply the lateral wall of the left and right ventricle, respectively.

Given this configuration, embolization of secondary branches of epicardial vessels such as marginal, septal, and diagonal branches only resulted in functionally inconsequent infarctions at the cost of complicated procedures while, contrariwise, embolization of the LAD was associated with much too extensive myocardial damage. We thus elected to focus on the dominant vessel; however the high sensitivity of the rabbit to ischemia required carefully controlling the level of its occlusion to ensure a substantial impairment of LV function, but with an acceptable mortality rate. Targeting a decrease in LVEF of 20% from baseline targeting was found to be a reasonable objective which could then be reproducibly achieved by locating the occlusion site at the mid segment of either the right coronary or the circumflex coronary artery.

These screening experiments then set the stage for the comparison of different infarction-inducing techniques which, to our knowledge, have not yet been reported. So far, the few studies which have used a closed-chest approach for inducing MI in rabbits have relied on the use of coils [5, 23]. We thus started our experiments with this type of material but, in our hands, neither free nor interlocking coils could generate reproducible results. It was difficult to load free coils into the delivery catheter and to control the exact site, timing, and extent of their deployment in the target artery and it was equally challenging to release interlocking coils in such a way as to occlude to coronary resulting in a substantial impairment of LV function that was still compatible with survival of the rabbit. The high cost of coils is another limiting factor. Both the release of gelatin sponge and the balloon inflation then looked initially attractive because of their purported simplicity. Indeed, gelatin sponge was plastic enough to yield an excellent conformability within the catheter but featured several disadvantages such as difficult handling, adherence to the catheter inner lumen, and poor visualization under fluoroscopy. Balloon-induced vessel occlusions, in turn, were arrhythmogenic unless inflations were kept short, in which case large infarcts could not be generated. Inspired by alcohol septal ablation performed in patients with hypertrophic obstructive cardiomyopathy, we finally tested alcohol injection. In contrast to the other techniques, alcohol injection allowed assessment of the TIMI flow grade just after the injection without removing the device. Because of the lack of selective injections, the first attempts performed with the microcatheter resulted in massive infarction. Optimized injections were then made possible using a coaxial total chronic occlusion balloon catheter as the microcatheter to achieve a superselective injection of 0.2 mL of alcohol. Considering both the amount of pathologically damaged myocardium, the resulting loss of echocardiographically measured pump function, and the survival of animals, this technique proved to offer the best risk to benefit ratio.

We acknowledge several limitations of the present study. Coils which were tested were those commercially available and we cannot exclude that customizing these devices would not have resulted in better outcomes. Second, functional assessments only consisted of gross measurements of EF and a more detailed analysis of the patterns of regional contraction and relaxation is clearly required to better characterize the hemodynamic effects of any coronary artery occlusion technique. Finally, the damage induced by alcohol injection may not accurately model that seen in patients with ischemic heart disease, although alcohol injection was found to depress LV function to a sufficient extent as to give room for detecting treatment effects for testing interventions. Despite these caveats, the present data can provide a useful benchmark for improving catheter-based techniques of coronary artery occlusion in rabbits and facilitate a broader use of this model for testing drugs or interventions designed to mitigate the consequences of ischemically induced LV dysfunction.

## 5. Conclusion

By following a stepwise approach, a minimally invasive, effective, and reproducible rabbit model of catheter-induced myocardial infarction has been developed which addresses the limitations of rodent experiments while avoiding the logistical and cost issues associated with large animal models.

## Figures and Tables

**Table 1:** Distribution and follow-up depending on embolization site.

Embolization site (*n* = )	Intraoperative events	3-week survival (*n* = )	Echographic assessment	Histologic assessment
LAD				
Proximal segment (1)	VF	0	—	Transmural infarct
Mid segment (2)	VF	0	—	Transmural infarct
Distal segment (2)	ACD	0	—	Transmural infarct
S. branch vessel (1)	—	1	No LV dysfunction	Nontransmural infarct
CA				
Proximal segment (1)	NSVT, CHF	0	—	Transmural infarct
Mid segment (2)	NSVT	2	LV dysfunction	Transmural infarct
Distal segment (0)	—	—	—	—
S. branch vessel (2)	—	2	No LV dysfunction	Nontransmural infarct
RCA				
Proximal segment (1)	CHF	0	—	Transmural infarct
Mid segment (2)	NSVT	2	LV and RV dysfunction	Transmural infarct
Distal segment (0)	—	—	—	—

LAD: left anterior descending; CA: circumflex artery; RCA: right coronary artery; LV: left ventricle; RV: right ventricle; VF: ventricular fibrillation; ACD: atrioventricular conduction disorder; NSVT: nonsustained ventricular tachycardia; CHF: congestive heart failure; S. branch vessel: secondary branch vessel.

**Table 2:** Distribution and follow-up depending on embolization device.

Embolization device (*n* = )	Intraoperative events	3-week survival (*n* = )	Echographic assessment	Histologic assessment
Free coil				
CA (3)	VF	0	—	Transmural infarct
RCA (1)	VF	0	—	Transmural infarct
Interlocking coil				
CA (2)	VF	1	LVEF decrease <15%	Nontransmural infarct
RCA (2)	VF	1	No dysfunction	No infarct
Gelatin sponge				
CA (3)	—	3	No dysfunction	Nontransmural infarct
RCA (1)	—	1	No dysfunction	No infarct
Balloon occlusion				
CA (2)	NSVT	2	No dysfunction	No infarct
RCA (2)	NSVT	2	No dysfunction	No infarct
Alcoholization				
CA (5)	VF	4	15% decrease in LVEF	Transmural infarct
RCA (3)	CHF	2	RV dysfunction 15% decrease in LVEF	Transmural infarct

CA: circumflex artery; RCA: right coronary artery; LV: left ventricle; RV: right ventricle; VF: ventricular fibrillation; NSVT: nonsustained ventricular tachycardia; CHF: congestive heart failure.

**Table 3:** Ultrasound assessment of alcoholization group.

	Before embolisation	After embolisation	*F*	Prob. > *F*
DIVS (mm)	3.8 ± 0.2	4.0 ± 0.2	2,354	0,155
DLVID (mm)	17.4 ± 0.4	20.1 ± 1.1	29,236	<0,001
DLVPW (mm)	5.5 ± 0.2	5.5 ± 0.3	0,052	0,823
SIV (mm)	4.1 ± 0.2	4.1 ± 0.2	0,220	0,648
SLVID (mm)	12.1 ± 0.5	16.5 ± 0.9	104,824	<0,001
SLVPW (mm)	5.7 ± 0.2	5.7 ± 0.2	0,018	0,894
LVEF (Teichholz (%))	58.0 ± 2.9	43.5 ± 1.6	113,648	<0,001
FS (%)	30.5 ± 1.8	18.0 ± 0.9	240,384	<0,001
LVESV (mL)	2.9 ± 0.2	3.4 ± 0.1	19,736	0,001
LVEDV (mL)	1.2 ± 0.2	1.8 ± 0.2	37,097	<0,001
LVEF (biplane Simpson (%))	58.3 ± 2.6	47.0 ± 2.4	60,8421	<0,001
SV (mL)	1.7 ± 0.2	1.6 ± 0.2	1,680	0,223

DIVS: diastolic interventricular septum; DLVID: diastolic left ventricle inner diameter; DLVPW: diastolic left ventricle posterior wall; SIVS: systolic interventricular septum; SLVID: systolic left ventricle inner diameter; SLVPW: systolic left ventricle posterior wall; LVEF: left ventricle ejection fraction; FS: fractional shortening; LVESV: left ventricle end systolic volume; LVEDV: left ventricle end diastolic volume; SV: stroke volume.
